# Quantitative Characterization of *Arnicae flos* by RP-HPLC-UV and NIR Spectroscopy

**DOI:** 10.3390/foods8010009

**Published:** 2018-12-24

**Authors:** Daniela Ivanova, Vera Deneva, Dimitrina Zheleva-Dimitrova, Vesela Balabanova-Bozushka, Daniela Nedeltcheva, Reneta Gevrenova, Liudmil Antonov

**Affiliations:** 1 Institute of Organic Chemistry with Centre of Phytochemistry, Bulgarian Academy of Sciences, Sofia 1113, Bulgaria; daniela.ivanova.91@gmail.com (D.I.); veradeneva@gmail.com (V.D.); dantonova@orgchm.bas.bg (D.N.); 2 Faculty of Pharmacy, Medical University of Sofia, Sofia 1000, Bulgaria; dimizheleva@gmail.com (D.Z.-D.); vbalabanova@pharmfac.net (V.B.-B.); rgevrenova@pharmfac.net (R.G.)

**Keywords:** *Arnicae flos*, near-infrared (NIR) spectroscopy, derivative spectra, high performance liquid chromatography (HPLC)

## Abstract

The possibility of applying near-infrared (NIR) spectroscopy to monitor 13 active components (phenolic acids, flavonoids, and sesquiterpene lactones) in *Arnicae flos* was studied. The preprocessing of the spectra were performed by using the conventional Golay-Savitzky procedure and the newly developed step-by-step filter. The results obtained show that the step-by-step filter derivatives provide a better signal-to-noise ratio at a lower convolution window. Better calibration for the content of protocatechuic acid, chlorogenic acid, caffeic acid, p-cumaric acid, ferulic acid, isoquercitrin, and quercetin were obtained by step-by-step filter derivatives, compared to the direct raw spectra processing and the Golay-Savitzky approach. Although the step-by-step filter substantially reduces the spectral distortion, the convolution procedure leads to loss of spectral points in the red end of the spectral curve. Probably for this reason this approach shows better calibration only in seven of the monitored 13 active components.

## 1. Introduction

The use of herbal medicinal products (HMP) is becoming increasingly relevant for modern healthcare as an alternative to conventional medicine [1]. Plant substances are typically characterized by a diverse composition, which can vary greatly according to the conditions and manner of cultivation, harvesting, processing and, storage [2]. In order to achieve reproducible quality and safety of the HMP, the raw materials from which they are harvested should be subjected to a comprehensive qualitative and quantitative analysis to ensure their authenticity and compliance with the pharmacopoeial requirements [3].

The subject of this paper is the plant substance *Arnicae flos* obtained from the species *Arnica montana* L. (mountain arnica) and *Arnica chamissonis Less. (Asteraceae)*. It is widely used in herbal and homeopathic medicine as an anti-inflammatory agent for external use on sprains, hematomas and for arthritic pain [4]. The pharmacological effects of the substance are due to a complex of active ingredients, the most important of which are the sesquiterpene lactones (STL) [5,6] and the phenolic compounds [5,7,8]. In 1998, Lange et al. [9] evaluated the use of *Arnicae flos* in Europe at over 50,000 kg of dry substance, and according to Franke et al. [10], over 20,000 kg of dried flowers are required to cover the annual needs of the German market alone. Today, the raw material is harvested both from wild populations and from cultivation.

The tremendous importance of mountain arnica for the pharmaceutical market and the variations in composition and pharmacological activity, require the use of reliable analytical techniques for qualitative and quantitative characterization and high-speed monitoring of the raw material. In the present paper, we aim to investigate the prospect of applying a non-destructive near-infrared (NIR) method for the rapid quantification of pharmacologically relevant components (sesquiterpenic lactones and phenolic compounds) of *Arnicae flos* using high performance liquid chromatography (HPLC) as a reference method. In the chemometric processing of the spectral data, along with the traditionally used Golay-Savitzky differentiation procedure, [11,12] we use a newly developed “step by step” filter [13] that substantially reduced spectral distortion. To our best knowledge, this is the first comparative study of these two pre-processing approaches in the investigation of dried medicinal plants.

## 2. Materials and Methods 

### 2.1. Sample Material

The experiment encompasses samples of the plant substance *Arnicae flos* with diverse origins: Two Bulgarian, one Polish cultivated collection, three cultivars, three botanical garden collections, one purchased from a pharmacy store and one from a wild population (Table 1). The extracts, as well as the NIR-spectra, were prepared using whole inflorescences including the involucral bract, which were shade-dried at room temperature and ground to a particle size of 2 mm.

### 2.2. HPLC-Analysis

In the current study, HPLC analysis was used for quantitation of phenolic compounds and sesquiterpene lactones. However, it should be noted that some alternative methods can be found described in the literature [14,15].

The chromatographic analysis was conducted on an HPLC-system produced by Varian (Varian, Inc., Walnut Creek, CA, USA) comprising of: Tertiary pump model 9012, Rheodyne manual injector with an injecting volume of 10 µm, and a UV-vis detector model 9050. The chromatographic columns used are as follows:

- For the phenolic compounds—Hypersil ODS C18, 5 μm, 250 × 4.6 mm I.D. (Shandon, Runcom, England), with precolumn 30 × 4.6 mm (Interchim, Montluço France) with the same adsorbent.
- For the sesquiterpene lactones—Luna 5 μm C18 100 A, 150 × 4.6 mm (Phenomenex, Torrance, CA, USA), with precolumn 30 × 4.6 mm (Interchim, Montluçon, France) with the same adsorbent.

The registering and treatment of the chromatographic data was conducted using Varian Star Chromatography Work Station software (Version 4.5, Varian, Palo Alto, CA, USA).

The chromatograms for each sample were registered at wavelengths consistent with the absorption maximum of the studied compounds—310 nm for the phenolic acids, 360 nm for the flavonoids and 225 nm for the sesquiterpene lactones, respectively.

For HPLC, separation of the phenolic compounds used an optimized method, as already described [16]. The analysis of the sesquiterpene lactones was conducted using the following conditions: Flow rate of 1.0 mL/min; temperature 35 °C. Mobile phase composition (A—water; B—methanol) and linear gradients in respect of A were as follows: 0 min–55%; 22 min–50%; 35 min–40%; 37 min–35%; 40 min–15% and 45 min–40%.

The quantitative analysis of the phenolic compounds was performed using the external standard method. The content of flavonoid glycosides was calculated as isoquercitrin, for the flavonoid aglycones as quercetin, and for the phenolic acids using standard solutions of each corresponding acid. For the quantification of the sesquiterpene lactones, a standard solution of santonin (1 mg/mL) was used as an internal standard. The obtained data are collected in Table 2.

### 2.3. Spectral Measurements

Spectral data were recorded using a double beam JASCO V-570 UV-Vis-NIR (200–2500 nm) spectrophotometer (JASCO International Co, Tokyo, Japan), equipped with an ILN-470 (JASCO International Co, Tokyo, Japan) integrating sphere (200–2000 nm) for the measurement of the reflectance spectra of solid and powdered substances. For each sample, three replicates were measured after homogenizing at optimal instrumental conditions (scan speed—100nm/min, detector response—slow, resolution—1nm).

The first derivative spectra were calculated alternatively using Golay-Savitzky (GS) differentiation (filter window = 10 points, polynomial degree = 2) [11] and the step-by-step filter (SBSF) [17] (filter window = 2 points, polynomial degree = 3) [12]. The software used for this purpose is described elsewhere [17].

Spectral data from all samples were used in the intervals:

270–850; 907–2000 nm for non-derivate spectra,
270–850; 935–1920 nm for spectra treated with GS,
270–850; 935–1980 nm for spectra treated with SBSF,

in order to eliminate the scattering caused by the change of the detector from UV-Vis to NIR.

### 2.4. Data Processing

The trial version of the Unscrambler software (v 9.7, Camo, Trondheim, Norway) was used to obtain regression models of the components.

The small number of samples available for building the model necessitated that the whole number be included in the calibration set. The model was validated using the leave-one-out cross-validation method. The precision of the final model was evaluated by the square root of the correlation coefficient (R^2^) and RMSECV (root mean square error of cross-validation). The RMSECV was determined by removing one of the samples from the calibration set followed by recalculating the model on the remaining samples and eventually testing it on the sample that was left out. This was repeated on all of the samples and the results were averaged. RMSECV was calculated using the equation (1):

(1) $$\mathrm{RMSCEV}=\;\sqrt{\frac{\sum_{i=1}^{n}{\left({\hat{y}}_{i}-y_{i}\right)}^{2}}{n}}$$

where n is the number of the samples included in the calibration set; y_i_ is the reference value of the concentration for the i-th sample; ${\hat{y}}_{i}$ is the predicted concentration value for the i-th sample when the i-th sample is subtracted from the model.

One of the crucial parameters in the construction of the calibration model is the number of principal components (PCs). In a simple system of one substance, the number of PCs would reflect the concentration of the substance and the influence of external factors—temperature, instrument conditions, impurities, etc. In a complex system, such as plant material, which contains hundreds of individual substances, it can prove difficult to predict all the relevant influences and their impact on the resulting spectrum. Nevertheless, it is essential to choose the optimal number of PCs that reflect the majority of variations in the composition of the samples, excluding those resulting from random errors and fluctuations. As a rule, determining the optimal number of PCs can be done in two different ways:

- choosing the lowest value of RMSECV or
- selecting the lowest number of PCs by which the largest percentage of variation can be described

The best result was obtained by combining both of these approaches, with the RMSECV value being the decisive factor for choosing an optimal number of PCs. The influence of the number of PCs on the slope and the offset of the calibration curve was also evaluated.

**Figure 1:**
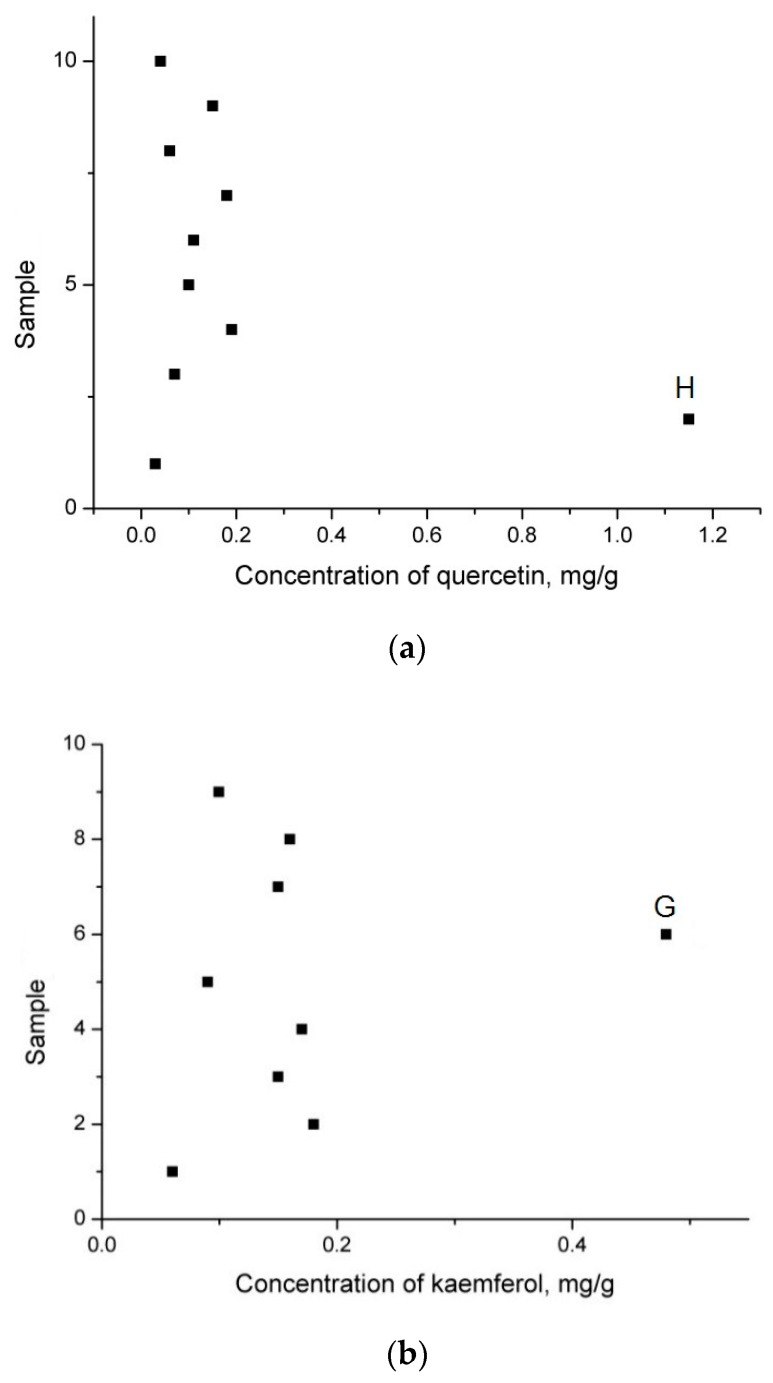
Distribution of the concentrations of quercetin (**a**) and kaempferol (**b**) in the samples *Arnicae flos*: H—origin Central America; G—origin Poland.

When selecting samples to be included in the calibration set, the variation of the y-axis reflecting the concentration of the components should also be taken into account. The calibration set should not only fully cover the range of values, measured for the whole set of samples, but also to exclude any outliers which introduce a deviation in the linearity of the model, making further applications to samples with an unknown composition unreliable. Therefore, it is very important for outliers to be detected before the construction of the model and removed from the calibration set. In the set of samples described in this paper, outliers were observed for the kaemphferol and quercetin concentrations in the samples (G) and (H) (Table 1) originating from Poland and Central America, respectively. This could be clearly seen on the graphical representation of quercetin (a) and kaemphferol (b) concentrations in the available samples (Figure 1). 

Before the construction of the calibration model, the spectral data were centered so that the absolute reflectance value for each wavelength in every individual spectrum was subtracted from the mean value at that wavelength for all samples. This procedure is beneficial in cases where the relative variation between samples is more important than the absolute variation. 

The final calibration models for all quantitatively determined compounds contained in *Arnicae flos*, were evaluated according to the minimum RMSECV value. The RMSECV values, R^2^ and, number of the major components are shown in Table 3. 

## 3. Results and Discussion

**Figure 2:**
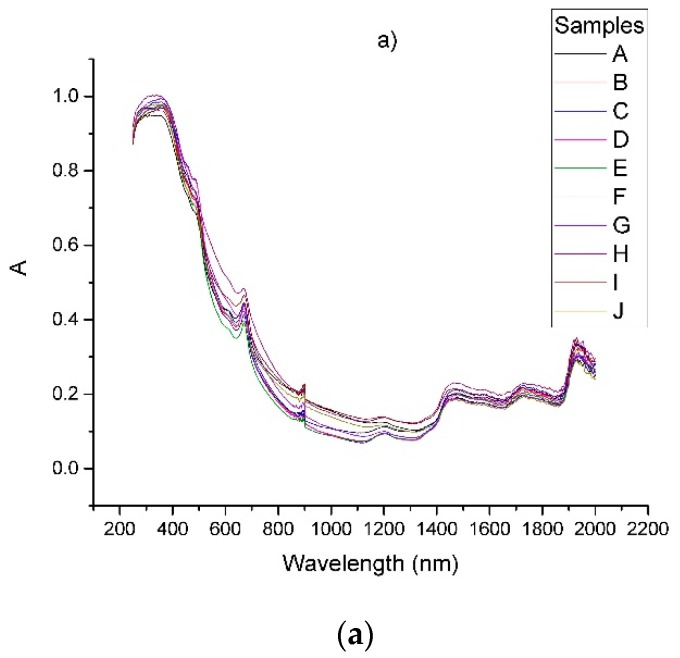

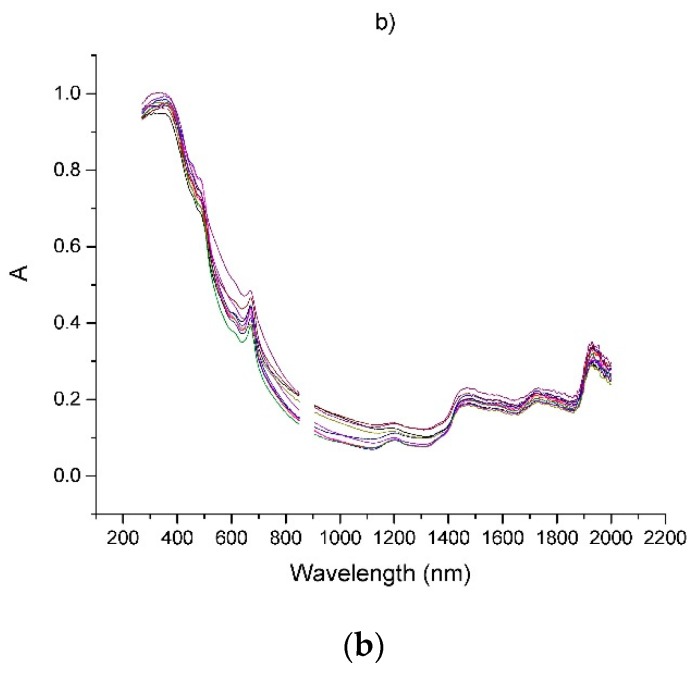
(**a**) Raw diffuse-reflectance spectra in the 250–2000nm range, obtained from dried *Arnicae flos*; (**b**) the processed spectra after removing ranges with detector switching and straight light area. The samples are defined in Table 1.

The raw spectra of the samples of *Arnicae flos* in the range 250–2000 nm are shown in Figure 2a. As can be seen, there are three broad peaks at 1920, 1720 and 1450 nm in the NIR region. Two peaks corresponding to the orange color (650 nm) of the petals and the green color (500 nm) of the involucral bracts are observed in the area of the visible spectrum. Basically, the visible region exhibits a relatively greater variation between the individual spectra as compared to the near-infrared one, due to the non-homogenic distribution of the powder particles of the samples and the presence of different plant parts. These variations were reduced substantially by measuring three replicates of each sample. In the region around 900 nm there is a sharp peak due to the change of the detector. This part of the spectra was removed to avoid spikes in the derivative curves. The same was done with the range 250–270 nm where the intensity of the sample led to saturation of the signal and to increased straight light. The corresponding curves are given in Figure 2b. It should be noted that the removal of these areas from the spectra significantly increased the quality of the derivative spectra and reduced errors in the model validation.

The raw spectra were preprocessed by using alternatively the Golay-Savitzky (GS) method and the step-by-step filter (SBSF). The parameters used (polynomial degree and number of points included in the filter window) were determined empirically according to the observed signal-to-noise ratio. It should be also taken into account that the convolution procedure used led to the loss of spectral data at the beginning and at the end of each spectral curve ((filter window-1)/2).

**Figure 3:**
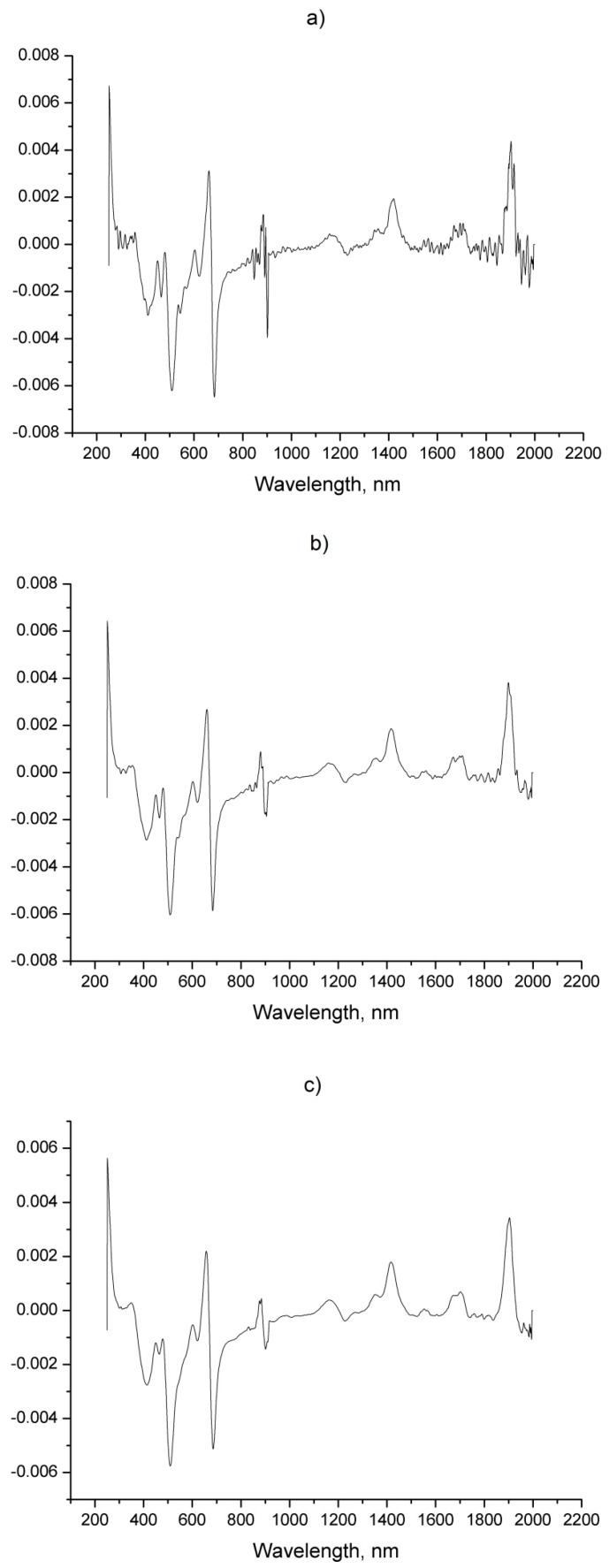
First derivative spectra obtained with Golay-Savitzky method: (**a**) 5-points; (**b**) 10 points; (**c**) 15 points filter window.

By varying the size of the filter window (Figure 3) when the GS method is applied with a filter window of 5 points the presence of sharp and narrow peaks, characteristic of noise patterns, is evident. Whilst a filter window of 15 points gave a good level of smoothing it also produced a decrease in the peak intensity resulting in the loss of information. Consequently, a filter window of 10 points was selected as an optimal compromise that preserved the maximum amount of useful information contained in the spectra, while lowering the noise level.

**Figure 4:**
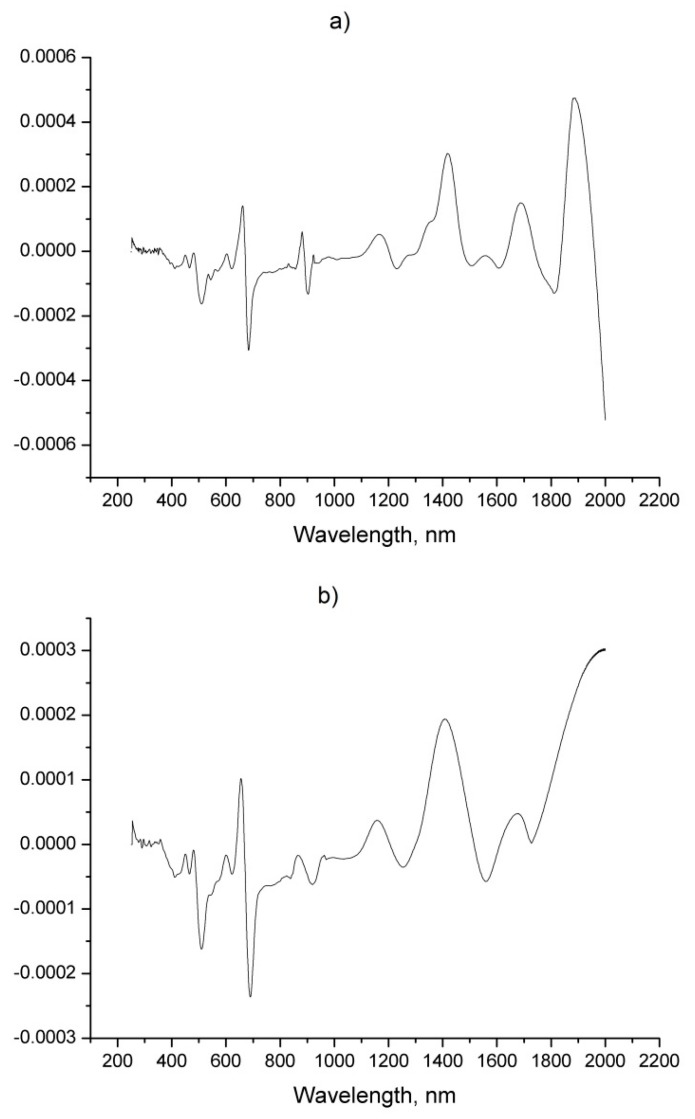
First derivative spectra obtained with SBSF (step-by-step filter) method: (**a**) 2-points; (**b**) 5 points filter window.

The calculation of the first derivative spectra using the SBSF requires a considerably smaller filter window, which allows a satisfactory smoothing of the spectrum while retaining its informativeness. When comparing two derivative spectra (Figure 4) produced with a filter window of 2 and 5 points, respectively, a significant improvement in the signal-to-noise ratio can be already be observed with a smaller filter window. Increasing the window leads to a loss of spectral information in the long-wavelength region. For that reason, a filter window of two points was selected as optimal, where no decrease in peak intensity in the long-wavelength region is observed, which compared to the GS-derived derivative spectra, is a key advantage in the further construction of the regression models.

**Figure 5:**
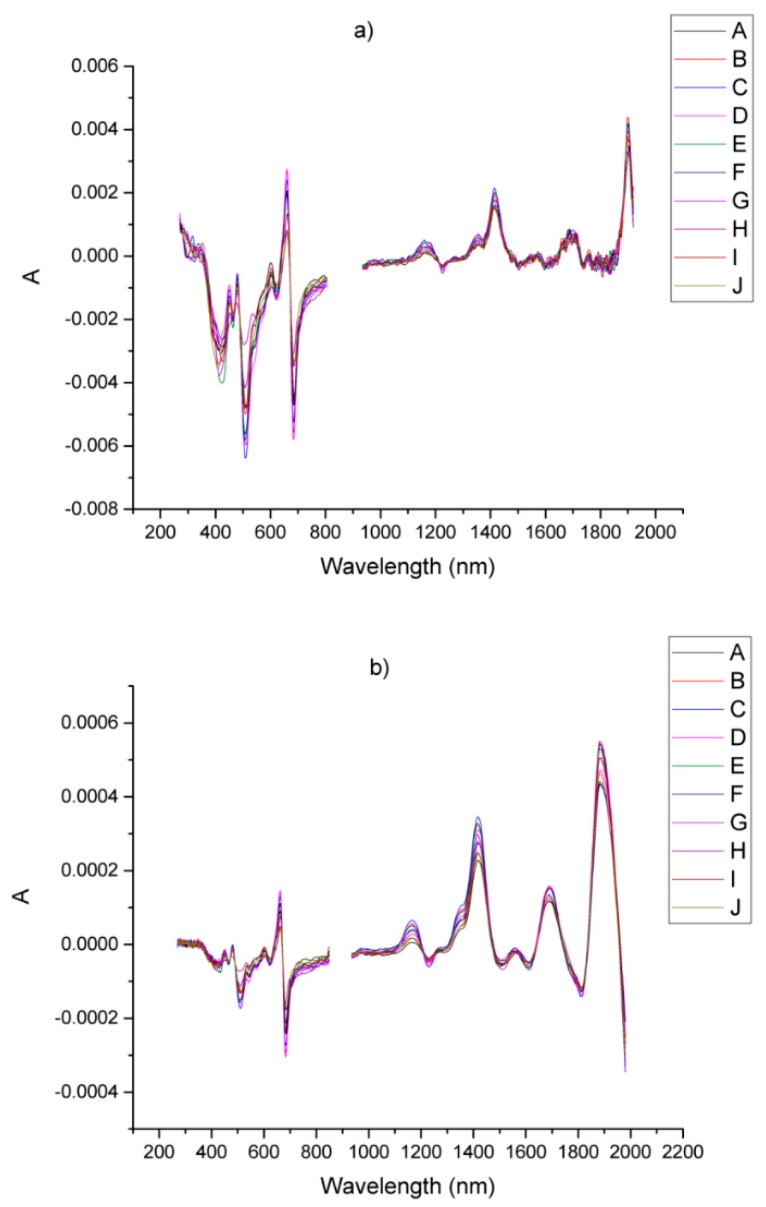
First derivative spectra of *Arnicae flos,* obtained from the curves from Figure 2b by using: (**a**) GS (Golay-Savitzky); (**b**) SBSF (Step-by-Step filter). The samples are defined in Table 1.

The direct comparison of the two preprocessing methods, made in Figure 4, very clearly shows that SBSF provides no attenuation in the near infrared area and maintains better sensitivity in this region compared to GS. The first derivative spectra obtained by both methods are shown in Figure 5.

The statistical parameters of the calibration models obtained by using both raw and first derivative spectra are collected in Table 3. As already described, due to the limited number of samples, the cross-validation method type leave-one-out was used for model validation. It is evident from Table 3, that nearly all tested compounds tend to have an equal or lower number of principal components in models built on the derivative spectra as compared to the raw ones. This is most likely due to the reduced influence of the noise and the effect of the baseline, achieved through the smoothing and the derivatization of the spectra.

**Figure 6:**
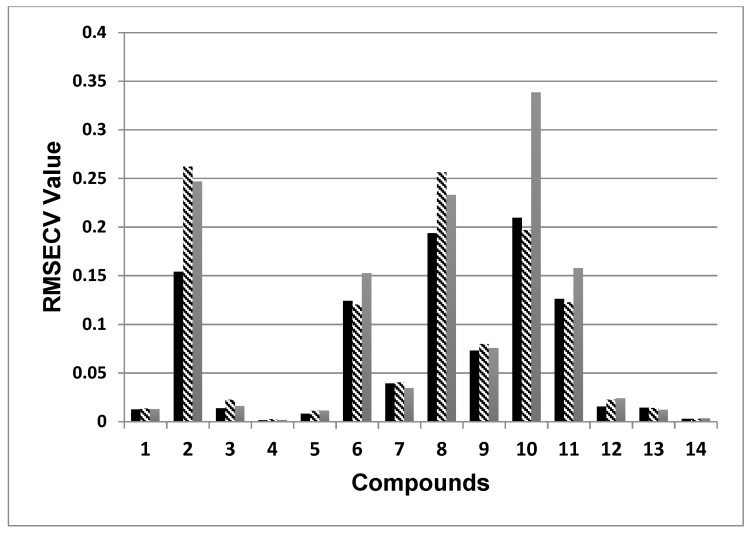
Comparison of the RMSECV values obtained from the different methods of spectral data processing: zero order curve—grey box; first derivative with GS method—striped box; first derivative with SBSF—black box. 1—protocatechuic acid, 2—chlorogenic acid, 3—caffeic acid, 4—p-coumaric acid, 5—ferulic acid, 6—sesquiterpene lactones, 7—14 flavonoids (7—isoquercitrin, 8—apigenin-7-glucoside, 9—astragalin, 10—isorhamnetin-3-glucoside, 11—quercetin, 12—luteolin, 13—kaempferol, 14—isorhamnetin); RMSECV—root mean square error of cross-validation.

Figure 6 shows a graphical comparison of the obtained RMSECV values (Table 3) for the individual methods with different types of spectral data processing. The smaller the value of RMSECV, the better the model describes the available data set.

The lowest values of RMSECV were obtained from the models built using SBSF for the following compounds: protocatechuic acid (1), chlorogenic acid (2), caffeic acid (3), *p*-cumaric acid (4), ferulic acid (5), isoquercitrin (8), and quercetin (12). Similar values were observed for the models built using SBSF and GS for the sesquiterpene lactones (6), apigenin-7-glucoside (9) and kaempferol (13). The best results for astragalin (10) and isorhamnetin-3-glucoside (11) were obtained from the GS derivatization of the spectra. 

Furthermore, for the sesquiterpene lactones (6), astragalin (10) and isorhamnetin-3-glu (11) a significantly higher error for the raw spectra were observed compared to the derivative ones. Presumably, this can be explained by the relatively strong effect of the baseline in areas where a large portion of the useful information about these compounds is contained. 

For some of the components (1, 2, 3, 7, 8 and 9), better results were obtained with the raw spectra than with the spectra processed with the GS method. This was probably due to the attenuation of the peak intensity of the longest wavelengths of the spectrum. Since these peaks contain much of the useful information of the spectral data, it is essential that they are maximally retained in the derivative spectrum. Therefore, in such cases, the SBSF proves more suitable and offers an advantage to the GS-method.

## 4. Conclusions

A comparative study of the applicability of two preprocessing techniques in UV-Vis-NIR spectroscopy, namely Golay-Savitzky (GS) smoothing/differentiation and the step-by-step filter (SBSF), for monitoring of the 13 active compounds in *Arnicae flos* was performed. Although SFBF shows some obvious advantages – better signal-to-noise ratio and lower spectra distortion at a low convolution window, it loses a substantial number of spectral points in the red end of the spectral curve. This is probably the reason this approach shows better calibration only in 7 of the 13 active components monitored.

## Figures and Tables

**Table 1:** Investigated samples origin.

Sample	Origin	Harvest Year
A	Bulgaria, Vitosha, cultivated	2012
B	Finland, Oulu University, cultivated	2002
C	Germany, agricultural cultivation, “Margurg” variety	2002
D	Finland Joensuu University, cultivated	2002
E	Finland, Turku University, cultivated	2002
F	Germany, agricultural cultivation, “Arbo” variety	2002
G	Poland, Lublin, University of Natural Sciences, cultivated	2011
H	Central America, bought from a herbal pharmacy	2010
I	Bulgaria, West Rodopi, cultivated	2013
J	Romania, Cluj, wild population	2013

**Table 2:** Content of the studies compounds in the samples listed in Table 1.

Compounds	Samples	A	B	C	D	E	F	G	H	I	J
1	C	0.020	0.099	0.11	0.098	0.080	0.140	0.013	0.069	0.120	-
	SD	0.006	0.001	0.002	0.003	0.007	0.011	0.001	0.013	0.010	-
2	C	1.48	1.030	1.5	2.06	1.51	1.39	0.680	1.66	0.79	1.80
	SD	0.10	0.001	-	0.17	0.36	0.07	0.004	0.87	0.12	0.22
3	C	0.073	0.066	0.061	0.079	0.062	0.162	-	0.017	0.03	0.106
	SD	0.012	0.001	-	0.040	0.042	0.076	-	0.002	0.02	0.037
4	C	0.064	0.048	0.058	0.050	0.058	0.052	0.056	0.045	0.050	-
	SD	0.005	0.006	-	0.005	0.003	0.002	0.003	0.001	0.002	-
5	C	0.096	0.096	0.062	0.075	0.062	0.046	0.071	0.015	-	0.0218
	SD	0.013	0.049	-	0.011	0.013	0.009	0.007	-	-	0.0130
6	C	1.44	1.27	0.77	1.15	1.18	1.17	0.86	0.25	1.73	0.93
	SD	0.30	0.03	0.18	0.15	0.08	0.14	0.16	0.06	0.21	0.07
7	C	1.49	1.49	1.92	0.93	1.82	2.12	1.93	1.74	0.27	-
	SD	0.23	0.09	0.03	0.04	0.45	0.12	0.02	0.19	0.03	
8	C	0.37	0.71	1.21	1.11	1.00	0.8	0.66	0.1	0.17	0.354
	SD	0.03	0.005	0.08	0.03	0.22	0.095	0.01	0.02	0.0001	0.18
9	C	0.83	2.52	2.5	3.37	2.34	2.86	1.58	-	0.44	1.197
	SD	0.07	0.29	0.001	0.29	0.51	0.35	0.02		0.03	0.05
10	C	0.43	1.03	1.22	0.93	1.833	1.08	0.75	-	0.22	0.604
	SD	0.08	0.008	0.008	0.052	0.376	0.128	0.009		0.01	0.018
11	C	0.15	0.19	0.11	0.07	0.06	0.1	0.18	1.15	0.03	0.0406
	SD	0.03	0.122	0.021	0.008	0.0001	0.061	0.006	0.0198	0.01	0.002
12	C	0.58	0.45	0.41	0.38	0.46	0.42	0.45	-	0.33	0.204
	SD	0.15	0.03	0.01	0.02	0.09	0.09	0.04		0.004	0.008
13	C	0.16	0.15	0.09	0.18	0.15	0.17	0.48	-	0.06	0.0995
	SD	0.02	0.02	0.01	0.02	0.04	0.007	-		0.004	0.004
14	C	0.028	0.022	0.025	0.026	0.035	0.033	0.053	-	0.023	0.0417
	SD	0.016	0.004	0.01	0.011	0.018	0.002	0.004		0.008	0.002

1—protocatechuic acid, 2—chlorogenic acid, 3—caffeic acid, 4—p-coumaric acid, 5—ferulic acid, 6 sesquiterpene lactones, 7—14 flavonoids (7—isoquercitrin, 8—apigenin-7-glucoside, 9—astragalin, 10—isorhamnetin-3-glucoside, 11—quercetin, 12—luteolin-7-glucoside, 13—kaempferol, 14—isorhamnetin); C—concentration in mg/g, SD—relative standard deviation.

**Table 3:** RMSECV, R^2^ values and, number of PCs determined during the construction of the calibration models.

No	Components	SBSF First Derivative			GS First Derivative			Zero Order (Figure 1b)		
		RMSECV	R^2^	PCs	RMSECV	R^2^	PCs	RMSECV	R^2^	PCs
1	Protocatechuic acid	0.0125	0.9153	7	0.0135	0.9020	9	0.0128	0.9116	5
2	Chlorogenic acid	0.154	0.8636	7	0.2621	0.6170	7	0.2467	0.6858	8
3	Caffeic acid	0.0136	0.8712	6	0.0225	0.7230	7	0.0161	0.8701	8
4	p-Coumaric acid	0.0014	0.9375	8	0.0024	0.8215	7	0.0018	0.9213	9
5	Ferulic acid	0.0081	0.9134	7	0.0111	0.8394	7	0.0113	0.8471	8
6	Sesquierpene lacones	0.1241	0.898	5	0.1204	0.9017	6	0.1525	0.8635	6
7	Luteolin-7-gly	0.0391	0.8471	8	0.0404	0.8344	6	0.0347	0.8774	9
8	Isoquercitrin	0.1936	0.9099	6	0.2561	0.817	4	0.2330	0.8187	10
9	Apigenin-7-gly	0.0729	0.9595	5	0.0799	0.9621	5	0.0756	0.9616	5
10	Astragalin	0.2094	0.9508	6	0.1967	0.9581	5	0.3840	0.8578	3
11	Isorhamnetin-3-gly	0.1262	0.9298	6	0.1227	0.9260	3	0.1578	0.9069	3
12	Quercetin	0.0153	0.9390	8	0.0224	0.8532	8	0.0239	0.8188	9
13	Kaempferol	0.0144	0.8608	6	0.0140	0.8837	3	0.0122	0.9240	8
14	Isorhamnetin	0.0027	0.9085	4	0.0027	0.9238	5	0.0034	0.8995	4

Step-by-Step filter (SBSF); correlation coefficient (R^2^); principal components (PCs); Golay-Savitzky (GS); root mean square error of cross-validation (RMSECV).
